# Drinking Water Supply in the Region of Antofagasta (Chile): A Challenge between Past, Present and Future

**DOI:** 10.3390/ijerph192114406

**Published:** 2022-11-03

**Authors:** Barbara Ruffino, Giuseppe Campo, Dafne Crutchik, Arturo Reyes, Mariachiara Zanetti

**Affiliations:** 1 DIATI–Department of Environment, Land and Infrastructure Engineering, Politecnico di Torino, 10129 Torino, Italy; 2 CleanWaterCenter@PoliTO, Politecnico di Torino, 10129 Torino, Italy; 3 Faculty of Engineering and Sciences, Universidad Adolfo Ibáñez, Santiago 7941169, Chile; 4 Departamento de Ingeniería en Minas, Universidad de Antofagasta, Antofagasta 1240000, Chile

**Keywords:** water scarcity, arsenic contamination, mining, Agua Potable Rural program, Chilean Water Code, climate change, water treatment plant, environmental sanitary engineering, SDG6: clean water and sanitation

## Abstract

Since the mid-nineteen century, when the first mining companies were established in the region of Antofagasta to extract saltpeter, mining managers and civil authorities have always had to face a number of problems to secure a water supply sufficient for the development of industrial activities and society. The unique features of the region, namely the scarcity of rainfall, the high concentration of arsenic in freshwaters and the increasing pressure of the mining sector, have made the supply of drinking water for local communities a challenge. In the 1950s, the town of Antofagasta experienced a serious drinking water crisis. The 300 km long aqueduct starting from the Toconce catchment, opened in 1958, temporarily ended this shortage of drinking water but created an even more dramatic problem. The concentration of arsenic in the water consumed by the population had grown by approx. ten times, reaching the value of 0.860 mg/L and seriously affecting people’s health. The water treatment plants (WTPs) which were installed starting from the 1970s in the region (namely the Old and New Salar del Carmen in Antofagasta and Cerro Topater in Calama, plus the two recent desalination plants in Antofagasta and Tocopilla), have ensured, since 2014, that the drinking water coverage in the urban areas was practically universal (>99.9%). However, the rural areas have continued to experience significant shortcomings regarding their capacity to ensure the quality and continuity of the water supply service in the long run. Presently, approx. 42% of the rural population of the region of Antofagasta does not have a formal supply of drinking water. The recent amendments to the Chilean Water Code (March 2022) and the interventions carried out in the framework of the Agua Potable Rural (APR) program were intended to reduce the socio-ecological inequalities due to the lack of drinking water in the semi-concentrated and isolated rural population.

## 1. Introduction

The development of society and industry all over the world is closely connected with the availability of water. The region of Antofagasta (Chile) has some unique characteristics that have always made the supply of water for local communities and industries a challenge. First of all, the region holds the driest desert area in the world, namely the Atacama Desert. In the region, the rainfall is very scarce, with only 1.7 mm/year in Antofagasta, the main town of the region. The historical shortage of freshwater is currently worsened by the effects of climate change [1]. Secondly, the superficial streams of the region are not only scarce, but are also of poor quality, due to high salinity and arsenic content. The main Chilean river, namely the Río Loa, contains concentrations of arsenic from 0.1 to 1 mg/L, with peaks as high as 50 mg/L in some springs located in the volcanic area where the river originates [2]. Finally, the Atacama Desert holds a major reserve of copper, lithium, molybdenum and natural nitrates [3]. In this context, the continuous growth of the extraction of minerals and metals has generated a significant economic benefit for the country, but, at the same time, it has increased the pressure on water resources. This problem causes a conflict between the mining sector, the other manufacturing sectors and local communities [4,5,6]. 

This paper presents and discusses the main problems related to the drinking water supply that have arisen in the region of Antofagasta in the past and in present days, and reviews the solutions that the government and civil authorities have found to cope with such issues. To the best of our knowledge, there are no other papers that comprehensively address the issues related to the drinking water supply—from the mid-nineteen century to present day—in the region of Antofagasta from the viewpoint of environmental sanitary engineering. 

After a description of the geographical and hydrological context of the region of Antofagasta (Section 2) and the consequent availability of water resources (Section 3), the paper presents a historical excursus of the criticalities related to the water supply in the area (Section 4). Section 5 and Section 6 review the developed solutions, with a special focus on sanitary issues, that is, the water treatment plants (WTPs), at the service of large (urban) (Section 5) or small (rural) communities (Section 6), which have been installed in the area. The performances of those WTPs are essential to ensure quality and continuity of the water supply service to the population in the long run. 

The results of the literature review reported in this paper are part of the activities carried out in the framework of the research project H2020-MSCA-RISE-2018–REMIND—Renewable Energies for Water Treatment and Reuse in Mining Industries, of which Politecnico di Torino (Turin, Italy), Universidad Adolfo Ibañez (Santiago de Chile, Chile) and Universidad de Antofagasta (Antofagasta, Chile) are partners.

## 2. The Geographical and Hydrological Context

The region of Antofagasta, namely Region II, is the second-largest region of Chile, comprising the three provinces of Antofagasta, El Loa and Tocopilla. It is bordered by the region of Tarapacá to the north, by the region of Atacama to the south and by Bolivia and Argentina to the east. The region of Antofagasta is located approximately between latitudes 21° and 26° south, being crossed by the Tropic of Capricorn, and between 71° and 67° west. The region covers an area of 127,221 km^2^, 85.2% of which is devoid of vegetation, while 14.3% are grasslands and shrubs, and 0.4% are wetlands [7].

The region has ten hydrographic basins, of which the Río Loa and the Salar de Atacama provide almost 90% of the surface water supply [8,9]. The Río Loa, located in the north of the region, with its 440 km, is the longest river in Chile and the only one in the region that flows into the sea. It has a contribution area of approx. 33,000 km^2^, with three sub-basins, namely Upper Río Loa, Middle Río Loa and Lower Río Loa, and a number of tributaries: Blanco, Chela, San Pedro de Inacaliri, Salado, San Salvador and Quebrada Amarga rivers. Similar to other rivers of the region, the Río Loa is not the product of the thaw, but it originates from the surfacing of underground water in the upper part of the basin. It receives contributions from groundwater through Palpan, San Pedro and San Pablo volcanoes in the Andes, at around 4000 m above sea level (a.s.l.) [10]. The Río Loa has an annual average flow of 0.55 m^3^/s in the fluviometric station “Río Loa before Represa Lequena”, located in the upper part of the basin (3315 m a.s.l.), that increases up to 1.4 m^3^/s in Yalquincha. At the river mouth, the flow averages 0.3 m^3^/s as a consequence of freshwater extraction and high evaporation [8,9].

The Salar de Atacama basin, located in the northeast of the region, is of the endorheic type, with a contribution area of approx. 16,000 km^2^. The water input is by snow and rain, and this water is then transported as surface water or groundwater. The major water supplies to the Salar de Atacama basin are from the Río San Pedro and Río Vilama, with a total estimated input of 1130 L/s. The Río San Pedro comprises the majority of the input, with a flow that varies from 679 to 900 L/s [10]. The Río Vilama is formed by the junction of the Puritana and Puripica thermal springs and has an independent network running parallel to the Río San Pedro. Both rivers have salty water. Water leaves the basin of the Salar as a result of evaporation, evapotranspiration or human activity. The annual average water recharge in the Salar is approximately 5 m^3^/s [10]. 

Other than the Río Loa and Salar de Atacama basins, there are other salt flats in the Andean zone of the region, with a predominance of small endorheic basins with small outcrops of groundwater that quickly infiltrate into alluvial deposits.

## 3. Water Demand and Water Availability in the Region of Antofagasta

Overall, Chile is a water-rich country, but water is unevenly distributed. The parameter “water runoff availability” relates the total freshwater resources with the total population in an area and indicates the pressure that a population puts on water resources. Chenoweth (2008) [11], basing his research on a sectorial and a development efficiency approach, individuated a minimum of 135 L per capita per day (equivalent to 49.3 m^3^/cap/year) for social and economic activities, which would permit the achievement of high human development. In the region of Antofagasta, the value of water runoff availability, equal to 52 m^3^/cap/year, is very close to the threshold compared, for example, to 190 m^3^/cap/year in the region of Atacama, 444 m^3^/cap/year in the Metropolitana Region or 169.500 m^3^/cap/year in the region of Los Ríos [12,13]. 

The water runoff availability only considers the availability of superficial or underground waters, but it does not consider the presence of dry periods or the quality of the water. Regarding the first point, rainfall in the northern regions of Chile is very scarce, with an annual mean precipitation of 1.7 mm in Antofagasta and 5.7 mm in Calama [9]. On the coast, precipitations occur in the winter months, unlike the upper part of the region (Altiplano), where the greater contribution to superficial waters and groundwater by precipitation is between December and March. The aquifers from which the main rivers originate have a very low recharge rate, which leaves them highly vulnerable to over-exploitation if the extraction rates are higher than recharge rates [14,15]. 

For a correct quantification of the water runoff availability, the quality of water is of capital importance, especially for the areas (such as Antofagasta and the regions located in the northern part of the country, namely Arica–Parinacota, Tarapacá and Coquimbo) where waters have very high values of electrical conductivity (EC). In these regions, the EC values range from approximately 1.5 to 7.5 mS/cm, which makes waters detrimental for crops and human consumption [12]. The water in the Río Loa is not only scarce, but also of poor quality due to high salinity. A recent study (2020), carried out by the Centro de Ecología Aplicada with the financial support of the regional government of Antofagasta, demonstrated that the salinity of the water has been gradually increasing along the river course, reaching the highest salinity levels in the lower areas [16]. The high salinity is related to both the origin of the river, that is the surfacing of shallow groundwater, and the intense evaporation that occurs in the Atacama Desert. Seasonal variations in salinity are also associated with irrigation in the Calama area, which causes leaching of saline soils [16]. The integrated diagnosis of the current environmental status of the Río Loa basin and its tributaries, with respect to the Integrated Vulnerability Index (which is composed of sub-indices of biota, water quality, river habitat and stressors), indicated a medium–high overall vulnerability [16].

Because of the facts described above, the region of Antofagasta is suffering a severe water scarcity, with a water demand that exceeds water supply by 22.1 m^3^/s [13]. This situation is emphasized by the effects of climate change. Chile has been in drought for 13 years and a recent OECD report (Water Risk Hotspots for Agriculture) ranks the country as 10th out of 142 subjected to more severe water risk [1]. The Directorate of Meteorology of Chile (DMC) estimates that, in 2050, the minimum temperature in northern Chile will increase by 2 °C and the rainfall will be reduced by 5–15% [1].

The water demand is expected to rise in the coming decades. In the region of Antofagasta, mining accounts for more than 65% of the gross domestic product (GDP) and has a very high impact on water demand (64%), being the main driver that causes the extremely high water-stress [15]. Mining uses more than 1 m^3^/s of water and, according to the Chilean Copper Commission, the region of Antofagasta will experience an extreme deficit in drinking water by 2025 unless urgent strategies are applied to reduce water requirements at mine sites and increase the awareness of the value of water [17]. In fact, although the riverbed of the Río Loa can recover if water consumption were drastically reduced, so far there are no measures in that direction, and mining companies have permits to extract more water than the little that is already flowing [16].

## 4. A Historical Excursus on the Water Demand in the Region of Antofagasta

Since the establishment of the first companies to extract saltpeter in the region of Antofagasta, around 1860, the continuous growth of mining activities has generated a significant economic benefit for the country, but it has inevitably increased the pressure on water resources. The first historical notes concerning the demand of water in the region of Antofagasta, for civil and industrial activities, date back to 1866. In that year, José Santos Ossa established a company to extract saltpeter from the mining site of Salar del Carmen. The water for mining activities was obtained from the Cerro Moreno [18]. Later on, the massive arrival of workers to the growing saltpeter office forced the company to obtain greater volumes of water. The first attempt at a solution was the construction of a condensing machine in 1868, namely Planta de destilación/desalación solar de Las Salinas. The characteristics and operating process of the machine were firstly described in 1883 in the Minutes of the Proceedings of the Institution of Civil Engineers [19,20]. The machine, which was quickly installed to supply the company’s tasks, was capable of delivering 270 m^3^ of water per day [21]. At the time, the population in Antofagasta was around 400 people, but it rapidly increased to 3000 in the beginning of 1872 with the discovery of the silver deposit of Caracoles. Water began to lack and its production became a good business [18]. In a few years (1870–1880), the number of condensing machines increased to 10 and they were mainly utilized to secure water for mining operations. To consume water, people had to go to the condensing machine, which were located close on the coast. The service was not immediate, with lines forming as people waited their turn. The distribution of water to the town was done in barrels transported in carts, or simply in barrels pulled by mules. However, despite the quality of the service provided by the plants, the town continued growing; the installed smelters were more and more productive and the demand for water increased, making the supply more difficult every day. The water available for household activities became expensive and of poor quality, thus determining the diffusion of infections and health diseases [22]. 

The situation continued until 1892, when a 340 km long pipeline, which withdrew water from the Río San Pedro, was opened. The pipe was originally drawn to feed the locomotives running along the railway line between Calama and Antofagasta. The Chilean State reached an agreement with the owner of the railroad, namely the Antofagasta and Bolivia Railway Company (FCAB), that obtained the concession to supply the town of Antofagasta and the intermediate points with water. However, the distribution network could not reach the whole community, which, that year, amounted to 13,500 inhabitants; for this reason, resellers of piped water soon emerged [21]. 

With the expansion of the mining sector and the consequent growth in the population, from the late nineteen century to the 1940s, the availability of water for civil uses progressively decreased [22]. In 1947, Antofagasta had a daily water deficit of 2000 m^3^ [23]. Until 1957, the town was supplied only with the water from the FCAB, which was sufficient for no more than two hours a day of service [18]. This lack of water, which negatively affected the life and the economy of the town, occurred for two main reasons: on the one hand, because population had increased, and on the other hand, because the resources obtained from the sale of water were not sufficient to improve the catchment and distribution network. In those years the FCAB had offered the government to increase the water capacity of the hydraulic infrastructure, thus securing a continuous supply for 30 years, in exchange for increasing the sale price. However, the government’s answer was negative, because an increase in the water sale price would have been very badly received by the population [23]. In such circumstances, the population began to present their complaints to the authorities of the central government of Gabriel González Videla, which agreed to open a new adduction on the Río Toconce [18]. 

By order of the government, the Directorate of Hydraulic Works (Dirección de Obras Hidráulicas, DOH) of the Ministry of Public Works (Ministerio de Obras Públicas, MOP) began the study for the positioning of the new adduction. The technicians from Santiago estimated that it was cheaper and more convenient to use centrifuged cement tubes, instead of those made of cast iron. However, the authorities of the Center for Progress and the Municipality of Antofagasta considered such a choice not suitable for a desert area. After a long and tedious controversy between the two parties, the construction of cement tubes was decided because, in this way, the work would have been cheaper by $800,000,000 [18]. From 1950 to 1952, four factories of reinforced cement pipes were installed but several problems started arising during the construction of the pipes, namely a cement shortage first, and subsequently, the way to join the pipes in order to build an aqueduct. Different glues were used, but none of them managed to bond tightly. Under pressure, they burst where the joint had been made. A field test demonstrated that the best glue was capable of offering resistance up to 80% of the pressure, but, when the pressure was increased to 100%, the suture broke. The test showed that centrifuged tubes were ineffective in an area where water dropped from more than 3000 m a.s.l. to sea level. When Don Carlos Ibáñez del Campo assumed the Presidency of Chile, he decided to reject the system based on cement pipes and to build the adduction with cast iron pipes [18]. 

In 1958, the works of the aqueduct ended and the waters of the Toconce catchment arrived in Antofagasta. The new aqueduct temporarily ended the shortage of drinking water but created an even more serious problem. Don Antonio Rendic was the first doctor who denounced what was happening. His patients exhibited nascent symptoms of cancer and the appearance of the skin indicated the presence of arsenic in the body. At first, the complaint of Dr. Rendic was dismissed by his colleagues but, some years later, the excess of arsenic in the drinking water was finally confirmed by the Medical Society. In 1968, President Eduardo Frei Montalva decided to establish arsenic abatement plants [18,23]. The first of them was built in the Salar del Carmen, just outside the town of Antofagasta, in 1970. That year, the maximum concentration of arsenic allowed in the water regulated through rule NCh409-1970 [24] was 0.120 mg/L.

## 5. The Problem of Arsenic and the Plants for Its Removal for Urban Areas

### 5.1. Origin and Concerns for the Arsenic Presence in the Region of Antofagasta

In the region of Antofagasta, water streams are characterized by concentrations of arsenic in the range of 0.1–1 mg/L, which is mainly due to natural causes, namely Neogene volcanic rocks and present-day geothermal activity in the Andes Cordillera [2]. The high concentration of arsenic in the volcanic rocks of the Neogene can be explained, given that the uplift and thickening of the continental crust in the Andes was associated with an enrichment of the lightest elements, namely arsenic, boron, potassium and lithium. The closure of the plateau and consequent generation of the endorheic basins starting in the Neogene, as well as the scarce precipitation, favored arsenic hyper-concentration [25]. 

The two areas with the highest concentration of arsenic in the basin of the Río Loa are (i) the confluence with the Río Salado, where the occurrence of arsenic is associated with chlorine, sodium and boron and (ii) the area of the river underneath the town of Calama, where arsenic is associated with sulfate and copper [10]. The chemical composition of water in the Río Loa is strongly influenced by the inflow of the Río Salado tributary, which originates from the geothermal field of El Tatio. The enrichment is observed downstream even at considerable distances from the confluence, through the important mining area of Chuquicamata, to the mouth [26]. The El Tatio geysers have one of the highest arsenic concentrations measured in hot springs worldwide, reaching values of 50 mg/L [25]. In this area, arsenic is mostly linked to carbonate. The mobility of arsenic might be due to multiple geochemical processes, including the chemical weathering of the mineral phases that incorporate sodium and bicarbonate ions into solution, with a consequent increase in pH and alkalinity [25].

Industrial processes associated with copper mining are another factor related to the presence of arsenic in the waters of the region of Antofagasta. In the last few decades, mining has been a driving force for the implementation of monitoring plants and mitigation strategies that contributed to coping with the problem of arsenic. Arsenic is a natural constituent in lead, zinc, gold and copper sulfide ores and can be released during the smelting process. The flue gases and particulates from smelters can contaminate nearby ecosystems downwind from the mining operation. However, it is difficult to assess the potential impact of mining activities on the arsenic content in the rivers of the region, because an arsenic baseline prior to mining activities has not been determined [10].

In the 1950s (1952–1957), the concentration of arsenic in the water supplied to the town of Antofagasta from the Siloli catchment, in the order of 90 μg/L, was not a concern [27]. In those years, no complaints or health problems were registered, neither in Antofagasta nor in the province of El Loa, where the inhabitants of Calama and other smaller towns (Quinchamale, Lequena, Concha, Lasana, Chiu Chiu) were supplied with water with an arsenic concentration of 0.21–0.23 mg/L [28]. In 1958, the Toconce catchment was opened and a more abundant water supply reached the town of Antofagasta through a 300 km long network of pipes. This new catchment determined an increase in the arsenic concentration in the water consumed by the population by approx. eight times, reaching a value of 0.860 mg/L [29,30]. The effects over time of such an arsenic concentration in water on people’s health were harmful, especially in children, who suffered from respiratory and cardiovascular diseases and dermal lacerations. Although, before 1960, the population of Antofagasta was concerned about other types of diseases, such as diarrhea, due to the shortage or poor quality of water, at the end of the 1960s, strong campaigns called attention to the danger of arsenic and the overexposure of the population. In 1970, the WTP of the Old Salar del Carmen was installed and the arsenic concentration in the water supplied to the population dropped sharply to approx. 0.110 mg/L [30,31]. 

Even though remediation processes had taken in place since 1970, carcinogenic effects followed arsenic exposure with long latency intervals, and the increased risks of As-related cancer (lung and bladder) remained for almost 40 years after the cessation of high exposure [32]. The lung and bladder cancer incidence rates in the population from Antofagasta, who had last been highly exposed in 1970, an average of 38 years before their cancers were diagnosed, were approx. from 4 to 7 times higher than those in people with low exposure [32]. Quite surprisingly, at the same time, a reduction in breast cancer mortality in the region of Antofagasta was observed after the exposure commenced and the trend reversed soon after the exposure was reduced in 1970 [28]. In fact, arsenic has been shown to cause induction of functional re-expression of the estrogen receptor in estrogen-negative breast cancer cells, which could make them less aggressive. 

### 5.2. Water Treatment Plants for Arsenic Removal Serving Urban Areas

The first WTP installed in the region of Antofagasta was the Old plant of Salar del Carmen in Antofagasta, after which other WTPs were constructed [31,33]. At present, the Aguas Antofagasta Group runs seven WTPs, operating in the region of Antofagasta, that serve urban areas and the largest towns (such as Antofagasta, approx. 350,000 inhabitants; Calama, approx. 140,000 inhabitants; Tocopilla, approx. 25,000 inhabitants; and Mejillones, approx. 10,000 inhabitants):

- Three WTPs, namely the Old (1970–year of starting operations) and the New (1989) plants of Salar del Carmen, located in Antofagasta, and the Planta de Filtros Cerro Topater (1978), located in Calama; all three WTPs are fed with river water that comes from the Alta Cordillera;
- One WTP in Taltal (namely O’Higgins, 1998) that treats the groundwater collected from the Agua Verde well field, located 70 km NE from Taltal;
- Two desalination plants located in Antofagasta, namely Desaladora Norte (previously called La Chimba, 2003) and in Taltal (2008);
- A new desalination plant located in Tocopilla that has recently (October 2020) started its operation.

**Figure 1:**
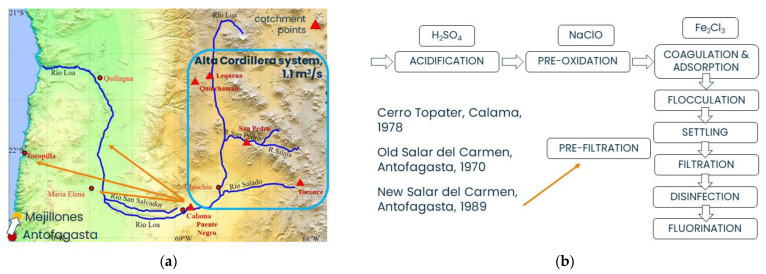
(**a**) Catchments points that feed the three WTPs located in Antofagasta (Old and New Salar del Carmen) and Calama (Cerro Topater); (**b**) Scheme of the treatment train of the three WTPs.

The Old and New WTPs of Salar del Carmen and the Cerro Topater WTP treat the same water, which comes from a mixing basin (“*estanque de mezcla*”) that receives the waters from five catchment points (see Figure 1a):

- Three of these (namely Lequena, Quinchamale and San Pedro) are on the Río Loa (Quinchamale and San Pedro are located on small influents/secondary rivers of the Río Loa, namely Quebrada Quinchamale and Río San Pedro, respectively);
- A fourth extraction point, named Toconce, is located on the Río Toconce, which is an influent of the Río Salado that is, in turn, an influent of the Río Loa. Together with the three previous catchment points, they form the Alta Cordillera system;
- The fifth extraction point is located just outside the town of Calama (namely Puente Negro), where the waters coming from the other four extraction points are mixed and then sent to the three WTPs. The last water source is of lower quality with respect to the waters coming from the Alta Cordillera and it is used only in case of emergency [34,35].

The Alta Cordillera system, through its four water sources, provides an average flow rate of 1.1 m^3^/s. 

Table 1 displays the quality of the waters coming from the five catchment points [34,35].

As can be seen from Table 1, waters from all five sources have arsenic concentrations far higher than the threshold value of 0.010 mg/L fixed by the in-force Chilean regulation (NCh 409-2005). Moreover, all waters have, on average, an alkali pH value not favorable for arsenic removal [30,31,33]. Turbidity in the waters coming from the sources located in the Alta Cordillera is generally low, but very high values can be found during the so-called “*invierno altiplánico*”, that is, the period from January to March. Table 1 reports the average maximum values of turbidity found in the waters collected from the three extraction points located in the Alta Cordillera during the “*invierno altiplánico*”. 

The three WTPs located in Calama and Antofagasta that are fed with river water make water suitable to be used for human consumption by using a series of treatments for solids, arsenic and pathogen removal. The three WTPs have a common treatment train that includes the phases of acidification and pre-oxidation, coagulation and adsorption, flocculation, sedimentation, filtration and final disinfection and fluorination (see Figure 1b) [34,35,36].

Acidification, conducted with sulfuric acid, is aimed at correcting the natural alkali pH of the raw water in order to improve the efficiency of the subsequent coagulation/adsorption phases, thus reducing the coagulant dose. The pre-oxidation is obtained by the addition of gas chlorine, which increases the oxidation state of arsenic from +3 to +5, thus making its removal easier [31]. Iron chloride (FeCl_3_) is used as a coagulant agent; it enhances the agglomeration of particles and generates iron hydroxide flocs that adsorb arsenic and determine its removal from water. Flocs are removed in a decanter/clarifier through a sedimentation process and a subsequent filtration with rapid filters [36]. The New WTP of Salar del Carmen can also count on a system of eight pre-filters. The pre-filters contain activated carbon and sand and are similar to the traditional filters used to remove turbidity. Finally, the filters contain high-density anthracite and sand, distributed above a gravel bed, and are characterized by filtration rates in the order of 130–150 m^3^/m^2^∙d [35]. These values are approximately one third–one half of the typical values used in rapid filters for the removal of suspended solids. In fact, arsenic is immobilized onto iron hydroxide flocs and a proper removal of arsenic, with the consequent achievement of the concentration value fixed by the legislation, can be obtained only when flocs are not perturbed or destroyed [35].

The combination of the afore-mentioned processes and their correct management allow the WTPs to achieve residual concentrations of arsenic in the order of 0.005 mg/L, well below the threshold value fixed by the Chilean regulation revised in 2005 (0.010 mg/L). In order to comply with the threshold concentration of arsenic, over the years, the Antofagasta and Calama WTPs have increased the dose of coagulant, namely FeCl_3_, used for the process up to values of 60 mg/L [37]. After the increase in the coagulant dose, an increased production of sludge in the decanters was observed and, at the same time, a better use of filters was observed, which resulted in requiring less frequent backwashing operations. In order to limit the working concentrations of coagulant and, consequently, its consumption, an acidification through the addition of sulfuric acid at the very beginning of the water treatment train in the three WTPs was introduced. In fact, arsenic removal through adsorption onto iron hydroxide flocs is enhanced by acidic pH values [36,37].

The water produced in the two WTPs of Antofagasta is distributed to the towns of Antofagasta and Mejillones. Conversely, the water produced in the WTP of Calama is distributed to Calama, Tocopilla and to the small centers located in the Pampa Salitrera (i.e., María Elena and the industrial centers of Coya Sur and Pedro de Valdivia) [34]. 

The O’Higgins WTP located in Taltal treats the waters collected from five wells from the well site of Agua Verde, with a treatment capacity of 32 L/s [37]. This WTP uses a direct filtration scheme to remove arsenic (approx. 0.070 mg/L, [33]) from the waters. The water extracted from the wells undergoes an oxidation by means of gas chlorine and an injection of coagulant, FeCl_3_, that generates iron hydroxide flocs able to adsorb arsenic; finally, flocs are entrapped in a rapid filter that contains sand and activated carbon [33]. The water treatment ends with a final disinfection with gas chlorine. In order to improve the efficiency in arsenic removal, several changes have been made to the WTP over the years [37]. The iron chloride used as a coagulant agent was supplemented with a polyelectrolyte and calcium hydroxide. The sand bed in the rapid filter was substituted with a resin and the number of filters was duplicated (from two to four). The Taltal WTP has a very limited capacity and supplies water to the town of Taltal only.

The towns of Antofagasta and Taltal are supplied with drinking waters that also come from two desalination plants. The two plants, namely the Planta Desaladora Norte in Antofagasta and the plant located in Taltal, have a treatment capacity of approx. 1000 L/s and 5 L/s, respectively. In these two plants, seawater undergoes a preliminary treatment of filtration and conditioning with chemicals to reduce phenomena of fouling and scaling and, subsequently, the main treatment of reverse osmosis (RO). The treated waters receive the final treatments of remineralization, disinfection and fluorination. The rejected water is disposed into the sea.

The Planta Desaladora Norte, the largest drinking water desalination plant in Latin America, was installed in Antofagasta with the goal of providing 100% of the drinking water required by the town [13]. The plant currently produces 91.24 ML/day (650 + 200 + 100 L/s) and supplies water to over 83% of the urban population [38]. The initial section of the Planta Desaladora Norte, with a capacity of 52,000 m^3^/d (650 L/s), was constructed in four phases, the first of which was put in operation in 2003 [39]. These four phases, with a capacity of 13,000 m^3^/day each, were delivered by the companies GS Inima (first three phases) and Atacama Water Technologies (final phase). The plant utilizes 8 + 1 intake pumps, delivering seawater from 350 m offshore and from a depth of 22 m. The pretreatment consists of 20 sand filters and 8 cartridge filters. Water is also conditioned with chemicals in order to reduce fouling and scaling before RO. The RO section has 8 racks of 90 pressure vessels with 7 elements per vessel. Each rack has one high-pressure pump and a Pelton turbine provides energy recovery. Twenty-four calcite chip filters provide permeate post-treatment [39]. To increase the plant treatment capacity, Atacama Water Technologies installed three new ROs skids (provided through Xylem), each producing 1000 m^3^/day. These new trains utilize the existing plant intake and the pre-treatment system. Each new train consists of 10 pressure vessels with 6 elements each and use Energy Recovery Inc. PX work exchangers [39]. 

Until 2020, the town of Tocopilla had been receiving water only from the WTP of Calama. In the case of problems to the water sources in the Alta Cordillera, to the Cerro Topater WTP or to the aqueduct from Calama to Tocopilla, the town could be deprived of water distribution. In October 2020 a new desalination plant started its operations in Tocopilla. It has a treatment capacity of 75 L/s that can be increased to 100 L/s. It can supply the town (>20,000 inhabitants) with 100% desalinized water. 

Desalination plants can guarantee a continuity of the drinking water service from an infinite source, thus generating a benefit for end-users. However, desalination faces three recurring issues, namely (i) water quality perception; (ii) environmental pollution due to the rejected water and (iii) high costs. An amount of 70% to 80% of citizens of Antofagasta are not satisfied with the quality of the drinking water, believing that consuming desalinated water is harmful to their health and definitely prefer bottled water [38]. The discharge of brine, that is, the desalination-rejected water, into stagnant environments can substantially increase the salinity and temperature of the receiving waters and, consequently, it can have an adverse effect on benthic organisms and seagrass beds. There is insufficient consensus regarding the influence of the Desaladora Norte plant on the marine biota. In fact, fishermen argue that marine life has decreased because of the desalination plant’s activities; conversely, the plant operators state that it has no impact [38]. Finally, a RO process has an energy demand between 0.5 and 4.0 kWh/m^3^, which varies depending on input salinity, temperature and employed technology [13]. This corresponds to approx. five times as much energy as traditional drinking water processes. The Desaladora Norte plant lacks an independent energy source; consequently, it must be supplied with energy from the national energy system, which is 63% based on fossil fuels [13,38]. 

## 6. Drinking Water Supply in Rural Areas: The Legal Framework and the Agua Potable Rural Program

### 6.1. Peculiarities of the Supply of Drinking Water in the Rural Areas of the Region of Antofagasta 

The installation of the WTPs described in Section 5 made the drinking water coverage in urban areas in 2014 practically universal, serving 99.9% of the population [40]. In 2010, Chile signed an agreement that formally recognized the human right to water and sanitation. Satisfying this right requires freshwater security, which is a well-recognized priority for human health, the environment and economy through Sustainable Development Goal (SDG) 6: clean water and sanitation. According to the World Health Organization and UNICEF, Chile is the Latin America country with the highest access to safely managed drinking water services [41]. However, the rural sector of the country has been continuing to show significant challenges regarding its capacity to ensure the quality and continuity of the water supply service in the long run. In fact, an amount equal to 47.2% of the rural population in Chile, and approx. 42% in the region of Antofagasta, do not have a formal supply of drinking water [42]. 

In Chile, there are two entities in charge of the formal supply of drinking water to the population, namely sanitation companies and the Agua Potable Rural (APR) program. The Aguas Antofagasta Group is the sanitation company operating in the region of Antofagasta. Conversely, the APR program was created in 1964 to cope with the reduced availability of drinking water in the rural areas of the country [43]. At that time, only 6% of rural households had access to drinking water, the shortage of which was the main cause of high rates of infant mortality [44]. Similar to other countries, Chile adopted the model of APR to provide infrastructure for drinking water supply to the most isolated localities. The program contemplated three components: (i) construction of the infrastructure of the water system with state funds; the local community is responsible for managing, operating and maintaining the system; (ii) rehabilitation and expansion of existing APR systems to improve deteriorated infrastructure and cope with growing demand—improvement in the continuity and quality of the service in terms of pressure in the network, water quality and losses reduction; (iii) strengthening of the APR committees and cooperatives through technical, financial and managerial advice [44]. However, the permanent decrease in water resources in many regions of the country has meant that complementary measures (i.e., informal supplies) must be implemented to maintain a supply of water for human consumption for families living in areas of deficit. On a country average, the informal supplies are 58.8% from wells, 25.8% from canals or springs, and 15.4% from cistern trucks. In the region of Antofagasta, an amount equal to 82% of the informal supply is from cistern trucks. The distribution frequency can range from daily to 7 or 15 days, depending on the accumulation systems and the distances from the different locations in the region. 

In 2018, a new drinking water regulation for cistern trucks was implemented, namely Supreme Decree n. 41. This arises from the need to regulate the conditions in which the distribution of drinking water is carried out through the use of trucks, in order to guarantee the supply of an innocuous product that ensures the health of the population. According to the regulation, the health authority must control some key aspects, namely the quality of the transported water from the origin to the final users through a series of physical, chemical and bacteriological parameters, the state of the pond, the availability of a chlorine analyzer, the route registry, the training certificate of the operators, and the quantity of water delivered to the users. 

Providing drinking water and sanitation to the population residing in rural areas is challenging because of the peculiar characteristics of rural areas, namely (i) the dispersion of dwellings; (ii) the geographical limitations for access to population; (iii) low socio-economic level; (iv) need of non-conventional technologies for the provision of services; (v) difficulty in offering technical assistance and training to service providers [40]. The access to secure water for the rural sector requires a governance with a clear mandate, as well as financial and human resources. Chile has one of the highest levels of fragmentation of responsibilities in water-related competences. More than 40 institutions are involved in delivering over 100 water-related functions. Within the MOP, several authorities have core competences over water management, including the DOH, the DGA, i.e., the General Directorate of Water (Dirección General de Aguas), and the Planning Directorate [1]. At present, an informal mechanism, that is, the Water Committee established in 2014, is responsible for the coordination between control and monitoring of water rights and execution of infrastructure. The Plan Chile 30/30 (i.e., the plan to reach USD 30,000 GDP (gross domestic product) per capita in 2030 through the development of infrastructures, namely transport, water, ports, etc.) offers an opportunity to combine perspectives and identify needs for water-related infrastructures [1].

In this framework, the existing Water Code published in 1981, that treated water as an economic good, was recently (March 2022) reformed. The reform had languished for a decade in the Congress and the recent severe decennial drought and massive protests in the autumn of 2019 forced lawmakers to rush through the long-delayed project. The main modifications to the Water Code concern Articles 5 and 6 and aim to prioritize human consumption and the environment. The changes made to Article 5 reinforced the character of water as a public good and stated that its domain and use belong to all Chileans, granting it a character of public interest to the protection of human consumption and sanitation [45]. In Article 6, it was stated that the right to water use will be granted through temporary concessions, in addition to allowing its renewal only if the availability and sustainability requirements of the supply source are met. The change to the existing law modifies the concept of water-use rights in order to give them a temporary character, prioritizing human consumption over other uses.

Furthermore, recently (2016), Law n. 20998 was promulgated to offer a specific regulatory framework, non-existent before then, to the supply of drinking water, the management of sewerage and the treatment of wastewaters in rural areas. Law n. 20998 was aimed at achieving a greater knowledge and control of the current APR systems and developing a long-term perspective in planning health services in rural areas [40]. The APR program had existed alongside the Water Code, even though the Code heavily differed from the solidarity model on which the APR program was based [41,46].

Initially, the objective of the APR program was to provide drinking water only to concentrated rural populations, that is, more than 150 inhabitants and 15 dwellings per km of water network. For concentrated rural populations, the full coverage was reached in 2010. Later on, the program started incorporating semi-concentrated and isolated/dispersed rural populations. However, over time, the APR systems have presented interruptions in the water supply, affecting approximately 350,000 people. The vast majority of these are unscheduled outages, which occurred in more than 40% of the APR systems of five regions (Valparaíso, 60%; Tarapacá, 51%; Arica–Parinacota, 46%; Antofagasta, 40% and Atacama, 40%) [42]. Between 2011 and 2014, an amount equal to 22.1% of unexpected maintenance and operation costs arose due to the deterioration of the APR systems [47]. This was mainly due to the uneven distribution of technical, financial and managerial skills across different APR committees and co-operatives. Although all committees have the obligation to use planning instruments such as annual financial statements and activity plans, many do not have them in practice [1].

### 6.2. The APR Systems in the Region of Antofagasta

In the region of Antofagasta, there currently are 12 working APR systems with an average age of 16.3 years [48] and 3 systems under construction or in a trial phase. The population density in the region is one of the lowest in Chile, with only approx. 5 inhab/km^2^. The number of inhabitants in rural areas supplied with APR systems is approx. 12,600, with a coverage of 57% [42]. Table 2 lists the APR systems present in the region of Antofagasta together with their main characteristics, namely number of distribution points and beneficiaries and employed technology [9,48]. As can be seen from Table 2, most of the APR systems are located in the Precordillera area, next to the comuna of Calama or San Pedro de Atacama. Only one APR system is located in the Costa area (Caleta Paposo, Taltal). 

Next to the comuna of Calama, in the area of “Desarollo Indigena Alto Loa”, there are six working APR systems, namely Chunchuri, Flor de Alfalfa, Caspana, Ayquina-Turi, Lasana and Chiu-Chiu. Chunchuri (Calama rural) is a south-western sector of the comuna of Calama, inhabited by one of the indigenous communities of the El Loa province. The APR system of Chunchuri only consists of a 25 m^3^ elevated tank and a connection to the water distribution network of the town of Calama through a 400 m PVC pipe. The supply system was put in operation in 2013 [49]. Before the execution of this work, the community had only precarious systems for the supply of water, that is, from cistern trunks or by carrying water to their homes with a bucket. In 2019–20, the APR system underwent conservation works for the installation of new meters, placement of cut-off valves to sectorize the distribution network and other measures aimed at strengthening the existing system [49,50]. Previously (2005), other rural sectors of the town of Calama (namely the Cerro Negro and Flor de Alfalfa) had received drinking water from the main network of Calama through the construction of a dedicated distribution network. In October 2021, the construction works of a new APR system in the surroundings of Calama, namely the Verdes Campiñas rural sector, were started. The project contemplated the connection of the new network to the point of the matrix at the intersection between Sotomayor and Pucón streets. The supply of water is proposed to be 140 L per inhabitant per day, with an average flow of 1.25 L/s distributed through HDPE pipes at 69 distribution points [51].

Caspana is a rural community with approx. 250 beneficiaries located in an indigenous area of the Alto Loa. In November 2020 the Regional Council of Antofagasta approved the proposal concerning the “feasibility study and design of the APR collection and treatment system in the Caspana locality”. Some delays in the realization of the works occurred because of an archeological find that has been under evaluation on the part of the Monumentos Nacionales [9]. No information was found on this particular APR system, but it is part of a program for the development of the areas inhabited by the indigenous population [52].

The APR system of Toconce started its operation in 2005. At that time, the APR system included a WTP, built by a Japanese company, where arsenic was removed thanks to the adsorption onto cerium oxide and/or iron hydroxide, Fe(OH)_3_, a storage tank, and a distribution network [53]. The construction works of the APR were supported by the comuma of Calama and the Japanese embassy. The plant did not work properly; no information is available concerning its present operational state. 

Ayquina-Turi, Paniri and Cupo are rural communities located in the El Loa province. They have been part of the APR program since 2016. In the site of these communities, the APR consists of a number of water ponds and a water network with 115 points of distribution. The water ponds are fed monthly by the municipality of Calama and the number of ponds can eventually be increased in situations of water scarcity. The communities have experienced episodes of severe water shortage, before the COVID-19 epidemic, during the Virgin of Ayquina holiday, during which the population triples or quadruples within a few hours [54], or, more recently, because of the pandemic contingency [55]. In response to the last occurrence in January 2021, Minera El Abra supplied each of the communities in the area with 10 m^3^ of drinking water. Recently (August 2021), the regional DOH and Codelco signed an agreement intended to design and put in operation appropriate technical solutions to provide drinking water to the communities of Cupo, Paniri and Ayquina-Turi [56].

**Figure 2:**
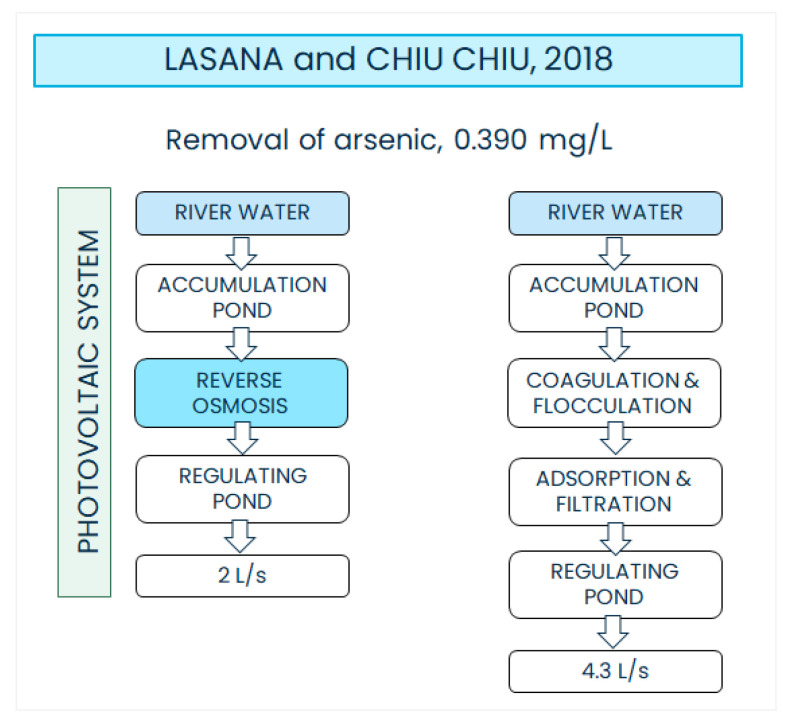
Scheme of the treatment trains for the APR WTP of Lasana (**left**) and Chiu-Chiu (**right**).

Since the year 2000, the town of Lasana has shared its APR system with the town of Chiu-Chiu. The APR system consisted of a WTP with a treatment capacity of 2 L/s, and included coagulation, flocculation, decantation and filtration units. The treatments were chiefly intended to the removal of arsenic from the waters of the Rio Loa, the average concentration of which was 0.390 mg/L. However, in recent years, a water supply of only 2 L/s for the two towns has proven to be insufficient because the populations in Lasana and Chiu-Chiu have increased. In 2017, Chiu-Chiu had a population of 650 inhabitants, which was projected to increase to 1300 inhabitants in the next 20 years. Consequently, in the same year, the Regional Council approved a project that was part of the measures for the development of APR systems in the region of Antofagasta. The project included the construction of a new WTP and the installation of pieces of equipment for the sectorization and better regulation of the existing water distribution network (cut-off valves, chambers and taps). Under the approved project, Lasana is projected to be supplied with drinking water coming from the new WTP with a capacity of 2 L/s and a RO module for arsenic removal. The electrical energy necessary for the new WTP is to be produced with 20 solar panels that allow for the reduction in energy costs for drinking water production. The old plant is proposed to be revamped and its treatment capacity increased to 4.7 L/s so as to cope with the water demand of the town of Chiu-Chiu (see Figure 2) [57,58]. 

In the area of “Desarollo Indigena Atacama La Grande”, there are five existing APR systems, namely those of San Pedro de Atacama, Río Grande, Toconao and Talabre, Socaire and Peine. 

**Figure 3:**
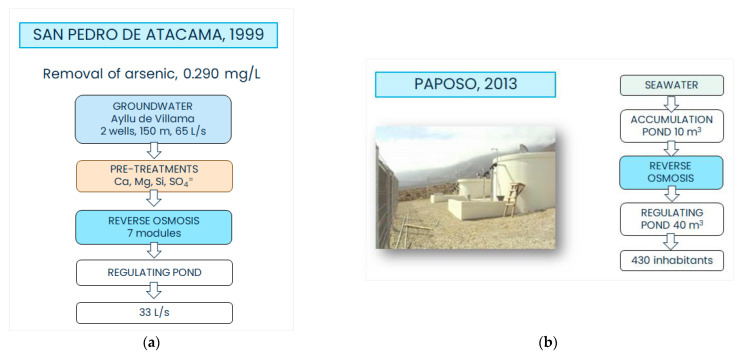
Scheme of the treatment train for the APR WTP of (**a**) San Pedro de Atacama and (**b**) Caleta Paposo, Taltal.

The APR system of San Pedro de Atacama started its operation in 1999 [59]. From 1999 to 2010, water was collected from the underground through a 150 m deep well (namely Vilama B-1) located three kilometers NE from San Pedro de Atacama. The initial authorized capacity of the Vilama B-1 well was of 40 L/s, which was increased to 80 L/s in 2008. A second well (namely Vilama B-2) was opened in 2010, but it started showing some problems immediately. In 2015, it was abandoned. Consequently, a third well (namely Vilama B-3) was opened that same year, with an authorized capacity of 40 L/s. In 2016, the overall flow rate of the water extracted from the well field of Vilama was 65 L/s (see Figure 3a). The groundwater is presently treated in a WTP that includes seven RO modules. An amount equal to 50% of the produced water does not have the quality required by Norm NCh 409-2005 and is intended for industrial uses [59,60]. 

Until 2015, the inhabitants of Río Grande in the comuna of San Pedro de Atacama, received waters from the Huaytiquina *vertiente* (spring), which is located about five kilometers from the town. The spring water was piped and stored in accumulation ponds, but no treatment processes were applied to it because it did not present any turbidity. In 2015, the Regional Development Fund together with Minera Escondida financed the construction of a new WTP based on RO technology, which was realized by HydroSolution. The intervention provides the inhabitants of Río Grande with water with characteristics that follow Norm NCh 409-2005 [61].

The APR system for the distribution of water to the communities of Toconao and Talabre in the comuna of San Pedro de Atacama started its operation in 2014. Previously, the two communities received water from the Quebrada de Silapeti (3 L/s), which already had a quality that complied with Norm NCh 409-2005, and from the Quebrada de Vilaco (6 L/s), the water of which contained a concentration of arsenic in the order of 0.017 mg/L, slightly higher than the threshold value fixed by the norm (0.01 mg/L) [62]. The APR system increased the flow rates of the two catchment points and introduced a WTP for the treatment of the water coming from Vilaco. The WTP consists of an abduction that, starting from the Quebrada de Vilaco, first reaches a chamber for sand removal, then a quartzite filter for the removal of fine particles and colloids and, finally, an ion exchange (IX) unit for arsenic abatement. Recently (October 2021), because of population growth and deterioration of the existing systems of collection, storage and distribution, the local authorities started the design of a plan for the modernization of the whole system of water treatment and distribution [62].

The APR system of Socaire, a town in the Norte Grande, was built in 2011. The WTP of the APR system included the units of filtration with a quartzite filter and RO. However, some problems in the WTP operation started appearing immediately after its start because of poor design, lack of coordination between the company in charge of the construction works and the APR committee, and cut offs in water and electricity supplies were frequent. Firstly, it appeared that the filtration unit was not only incapable of fulfilling its task, but it also concentrated the particles more, thus causing a rapid saturation of the RO unit that had to be frequently chemically cleaned. The company had to make changes in the construction so that the filtration unit could serve for the purpose for which it was intended. The WTP was also subjected to unexpected water cuts or power outages that affected the operation of the RO plant. Water cuts occurred because the water, which had to be sent to the WTP, was diverted and used to irrigate some areas of Socaire, thus leaving the WTP without supply. Agreements were reached so that the cuts were notified and, in the longer term, so that a diversion was built to share the water. Recently (February 2021), the MOP, through the regional DOH, carried out the conservation of the supply networks of the APR with the aim of increasing the useful life of the existing infrastructure. The conservation works included the supply and installation of a chlorination system, the maintenance of pressure chambers and taps, and the installation of new meters and new home starts for the 150 points of distribution and approx. 470 inhabitants who benefited from the APR system [63,64,65,66].

Peine is a town located 150 km south from the comuna of San Pedro de Atacama. No information is available concerning the characteristics of the APR system, which has 180 points of distribution for a total of approx. 560 beneficiaries [66]. Works for the conservation of the APR system were carried out from 2020 to early 2021 and included the maintenance or change of valves and meters and the construction of new hydraulic works [66]. 

Quillagua is an oasis located in the riverbed of the Río Loa, 70 km from the river mouth at the border between the regions of Antofagasta and Tarapacá. In 2002, National Geographic cataloged Quillagua as the driest point on the planet, with a record of 0.2 mm of rainfall over 40 years [67]. Until recently, the 160 inhabitants of Quillagua depended on the municipality of María Elena for the supply of water. The water was transported in tank trucks and stored in three tanks with an overall volume of 100 m^3^. In 2015, an investment was made for the construction of a WTP that used IX and RO technologies to make the water from the Río Loa drinkable. The Quillagua APR system included pipes and reinforced concrete tanks for raw water collection and storage, IX and RO units, chlorination booths, two evaporation pools for water rejected from the RO unit, and solar panels for energy supply. In March 2018, the WTP was operational in its trial run [68,69]. In May 2020, the project was 92% complete; the re-mineralization system remained to be installed and maintenance interventions were required for the reception chamber and the RO system [50]. In a recent work, Acuña and Tironi (2022) reported that the increase in the Rio Loa water discharge and change in water chemical composition, which occur during the “*invierno altiplánico*”, negatively affect the efficiency of the WTP, making it unusable for months. The rises in the river during the summer months (“*crecidas*”) are a threat to the infrastructure, and their muddy waters obligate the operators to let the WTP rest and cover the membranes with a preserving agent as long as the dirty water persists [67]. 

The APR system of Caleta Paposo is located in the town of Paposo, 54 km north from Taltal, in the Territorio Costa. The APR system is supplied with seawater through a drain with a barbican, which prevents the entry of sand into the catchment. Seawater is sent to a 10 m^3^ accumulation pond and, from there, to a RO unit that makes the water drinkable according to the Chilean standards (see Figure 3b). After membrane filtration, the water is pumped into a 40 m^3^ regulating tank for the subsequent distribution to the 144 outbreaks located along the distribution network to finally reach approx. 430 end-users. The plant started its operation in 2013 and presented some problems during the first five years of operation, but after the conservation works performed in 2018, it has been operating regularly. At the end of 2021, a new campaign of maintenance and renovation works, which included the maintenance of the remineralization system and of the clean-in-place equipment, as well as the implementation of the automation and control system in the WTP, was carried out [70].

Antofagasta is the only region in the country where the DOH is in charge of the technical assistance to the APRs [9]. Although the APRs may rely on the technical advice from the DOH, the advice is not always constant, especially in the matters of water treatment, which is why criticalities have arisen regarding the quality of the service and the quality of the water. In a survey carried out by the MOP and published in 2016, no information was found on the quality of the water being distributed by the APRs in the region [9]. Consequently, it cannot be compared with the existing regulation. This topic is relevant for the APR beneficiaries, since they are not aware of the quality of the water that they are consuming. Therefore, in order to reduce the criticalities on the APR service, it is necessary to improve the operation and maintenance of the systems with plans that aim to secure monitoring and control of water quality, continuity in the operation of infrastructures, as well as training for personnel in charge of the systems. 

**Table 2:** APR systems in the region of Antofagasta with their main characteristics (number of distribution points and beneficiaries and employed technology).

Area	Comuna	APR System	Year	Number of Distribution Points [9]	Number of Beneficiaries [9]	Technology
Precordillera	Calama	Chunchuri–Calama Rural	2013	98	295 [49] 400 [49]	Connection to the water distribution network of Calama
Precordillera	Calama	Flor de Alfalfa	2005	79	237	Connection to the water distribution network of Calama
Precordillera	Calama	Verdes Campiñas	>2021	69 [51]	580 [51] 863 (2040) [51]	Connection to the water distribution network of Calama
Precordillera	Calama	Caspana	>2020	189	250	N.A.
Precordillera	Calama	Toconce	2005	98 [53]	100	Adsorption onto cerium oxide/Fe(OH)_3_ (working?)
Precordillera	Calama	Ayquina–Turi	2016	115	150	Tank trucks from Calama (Project of a new plant? 2021)
Precordillera	Calama	Lasana	2017	117	431	RO, 2 L/s
Precordillera	Calama	Chiuchiu	2000	184	1200	Coagulation–flocculation, revamped, 4.7 L/s
Precordillera	San Pedro de Atacama	San Pedro de Atacama	1999	2800 [60]	6200 [60]	Groundwater, 7 modules of RO
Precordillera	San Pedro de Atacama	Río Grande	2016	38	120	RO
Precordillera	San Pedro de Atacama	Toconao	2014	350	1100	Quartzite filter + IX
Precordillera	San Pedro de Atacama	Socaire	2011	150 [64]	465 [64]	Quartzite filter + RO
Precordillera	San Pedro de Atacama	Peine	2009 (?)	180 [64,66]	558 [64]	N.A.
Precordillera	María Elena	Quillagua	>2020	20	120	IX + RO
Costa	Taltal	Caleta Paposo	2013	144 [70]	430 [70]	Desalination plant (RO)

N.A. Data not available? The authors are not completely sure of the reported information.

## 7. Conclusions

This study highlighted three main issues concerning the criticality in the supply of drinking water in the region of Antofagasta:

1. Even if Chile has quite a large availability of water, it is not evenly distributed over the country. Specifically, the region of Antofagasta is suffering a severe water crisis due to the unique characteristics of the area, namely the natural dryness, bad quality of freshwater and pressure of the mining industry. The water crisis is even expected to be worsened by the effects of climate change. The Río Loa is the most important water source in the region and in the Atacama Desert area. However, the environmental flow of the Río Loa, that is, the flow that allows the sustenance the ecosystem, as well as the means of subsistence and welfare of the people who depend on that ecosystem, is not sufficient to meet the demand for all uses with its current water flows and pressures. According to a recent diagnosis, a portfolio of projects that should be implemented to reduce the pressure on the Río Loa includes (i) the definition of water supply alternatives (ii) the recovery of sites of environmental relevance and (iii) the development of a more effective management of the territory [16];
2. Since the end of the nineteen century, large efforts have been made to construct water distribution networks and, in more recent years, WTPs to supply the large towns (such as Antofagasta, Calama, Tocopilla and Mejillones) and small centers with drinking water that complies with NCh409-2005. Further to these interventions, the drinking water coverage in urban areas in 2014 was practically universal, serving 99.9% of the population [40]. Especially in the urban area of the town of Antofagasta, the result was made possible after the installation of the Desaladora Norte desalination plant, the largest in the Latin America, with a treatment capacity of approx. 1000 L/s [13,39]. It can guarantee a continuity in the drinking water service from an infinite source, thus generating a benefit for the town. However, surveys carried out among the citizens have revealed that a large part of the population, between 70% and 80%, was not satisfied with the quality of the drinking water because of organoleptic issues related to taste, color and odor, or because they distrusted its direct consumption because of the strong communal memory of past diseases due to arsenic contamination [38]. Furthermore, some authors argue that the gradual introduction of desalinated water into the town’s metabolisms has exacerbated the existing socio-ecological inequalities, thus maintaining the perception of a situation of water scarcity, especially for low-income citizens [71]. Other criticalities concern the impact of the desalination plant on the marine biota, because of the discharge of the RO rejected waters into the ocean, and the lack of an independent energy source, which presently compels the plant to be run with the national energy system, which is 63% based on fossil fuels [38];
3. Supplying drinking water and sanitation to the population of rural areas remains a challenge and 42% of the rural population of the region of Antofagasta still does not have a formal supply of drinking water [42]. The APR program, created in the mid-1960s, contributed to providing rural water service infrastructures. In the region of Antofagasta, there are 12 operating installations and 3 systems under construction or in a trial phase [48]. The beneficiaries of the APRs are responsible for managing, operating and maintaining the system through the APR cooperatives and the support of the DOH. The problems attributed to the APRs in recent years are related to a number of factors, namely (i) the lack of a systematic and comprehensive monitoring of the operations carried out at the installations, (ii) the inadequacy of the chosen technology to the specific installation site, and (iii) low managerial skills and technical knowledge of the committees that are responsible for operating, maintaining and financing the APR systems [40,67].

## Figures and Tables

**Table 1:** Quality of the waters collected from the five catchment points that feed the Calama (Cerro Topater) and Antofagasta (Old and New Salar del Carmen) WTPs.

	NCh409-2005	Toconce	Lequena	Quinchamale	San Pedro	Puente Negro
		Alta Cordillera System				
Altitude, m a.s.l. ^a^	-	3335	3245	3056	3290	2284
Flow rate, L/s ^a^	-	610 ^c^ (470) ^d^	576 ^c^ (550) ^d^	400 ^c^ (300) ^d^	90	150 ^c^ (69) ^d^
As, mg/L ^b^	0.01	0.82	0.25	0.18	0.50	1.24
Cl, mg/L ^b^	400	135	163	429	235	2144
pH ^b^	6.5–8.5	8.17	8.37	8.00	8.64	8.14
Turbidity, NTU ^b^	2	1.015	6.450	4.782	NA	NA

^a^ from SiSS, 2016 [34]; ^b^ from CAUSSE, 2016 [35]; ^c^ design collection capacity; ^d^ nominal rights granted (“*derechos nominales otorgados*”) flow rate; NA, not available.
